# Micro‐Engineered Organoid‐on‐a‐Chip Based on Mesenchymal Stromal Cells to Predict Immunotherapy Responses of HCC Patients

**DOI:** 10.1002/advs.202302640

**Published:** 2023-07-23

**Authors:** Zhengyu Zou, Zhun Lin, Chenglin Wu, Jizhou Tan, Jie Zhang, Yanwen Peng, Kunsong Zhang, Jiaping Li, Minhao Wu, Yuanqing Zhang

**Affiliations:** ^1^ Zhongshan School of Medicine Sun Yat‐sen University Guangzhou 510080 China; ^2^ School of Pharmaceutical Sciences Sun Yat‐sen University Guangzhou 510006 China; ^3^ The First Affiliated Hospital Sun Yat‐sen University Guangzhou 510080 China; ^4^ The Third Affiliated Hospital Sun Yat‐sen University Guangzhou 510635 China

**Keywords:** cancer immunotherapy, drug screening, mesenchymal stromal cells, microfluidics, tumor organoids

## Abstract

Hepatocellular carcinoma (HCC) is one of the most lethal cancers worldwide. Patient‐derived organoid (PDO) has great potential in precision oncology, but low success rate, time‐consuming culture, and lack of tumor microenvironment (TME) limit its application. Mesenchymal stromal cells (MSC) accumulate in primary site to support tumor growth and recruit immune cells to form TME. Here, MSC and peripheral blood mononuclear cells (PBMC) coculture is used to construct HCC organoid‐on‐a‐chip mimicking original TME and provide a high‐throughput drug‐screening platform to predict outcomes of anti‐HCC immunotherapies. HCC‐PDOs and PBMC are co‐cultured with MSC and Cancer‐associated fibroblasts (CAF). MSC increases success rate of biopsy‐derived PDO culture, accelerates PDO growth, and promotes monocyte survival and differentiation into tumor‐associated macrophages. A multi‐layer microfluidic chip is designed to achieve high‐throughput co‐culture for drug screening. Compared to conventional PDOs, MSC‐PDO‐PBMC and CAF‐PDO‐PBMC models show comparable responses to chemotherapeutic or targeted anti‐tumor drugs but more precise prediction potential in assessing patients’ responses to anti‐PD‐L1 drugs. Moreover, this microfluidic platform shortens PDO growth time and improves dimensional uniformity of organoids. In conclusion, the study successfully constructs microengineered organoid‐on‐a‐chip to mimic TME for high‐throughput drug screening, providing novel platform to predict immunotherapy response of HCC patients.

## Graphical abstract

Coculture with mesenchymal stromal cells and peripheral blood mononuclear cells promote the growth of hepatocellular carcinoma organoids, mimicking the tumor microenvironment by supporting the monocyte/macrophage survival and M2 macrophage differentiation. The microfluidic chip is designed to achieve high‐throughput MSC‐PDO‐PBMC coculture and dynamic drug delivery, further displaying great potential in assessing immunotherapeutic response.

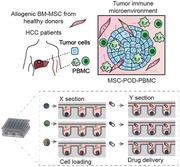

## 1 Introduction

Hepatocellular carcinoma (HCC) is the sixth most common cancer and the third leading cause of cancer‐related death worldwide.^[^ ^1^ ^]^ Immune checkpoint inhibitors (ICI) have been increasingly used in HCC treatment, but only 20% of HCC patients benefit from immunotherapy.^[^ ^2^ ^]^ Meanwhile, current approaches to select suitable patients for ICI treatment mainly depend on high PD‐L1 expression and lymphocyte infiltration.^[^ ^3^ ^]^ Still, prognosis prediction is often incorrect due to lacking an original tumor microenvironment (TME). TME mainly consists of immune cells, including macrophages and T lymphocytes, as well as stromal cells like cancer‐associated fibroblasts (CAF) and mesenchymal stromal cells (MSC, also called mesenchymal stem cells), which together influence tumor progression and host response to immunotherapy.^[^ ^4^ ^]^ Tumor‐associated macrophages (TAM) are the most abundant cells in the tumor immune microenvironment, which form ≈ 30% to 50% of immune cells, and play an important role in tumor metastasis, immunosuppression, and drug resistance.^[^ ^5^ ^]^ However, the in vitro proliferation and long‐term maintenance of macrophages remain challenging.^[^ ^6^ ^]^ In this regard, establishing an efficient drug screening platform that takes TME, especially TAM, into account has great importance in personalized cancer therapy.

Patient‐derived organoids (PDO) are 3D‐culture models which can proliferate in vitro and retain some key characteristics of the original tumor specimens,^[^ ^7^ ^]^ therefore displaying promising potential in application on drug screening and precision therapy.^[^ ^8^ ^]^ However, their potential to predict clinical outcomes in patients is still unclear, and the in vitro evaluation model for immunotherapeutic drugs remains lacking. Several problems exist in current techniques for PDO culture, limiting their application on personalized drug screening. It takes at least 3–4 weeks to culture PDOs from the HCC biopsy samples, and the success rate is only around 26–33%.^[^ ^9^ ^]^ During this long‐period culture, many cytokines are continuingly added into the culture media to sustain PDO proliferation, which is time‐ and cost‐consuming. Moreover, to reconstitute TME for drug screening, PDOs are often co‐cultured with peripheral blood mononuclear cells (PBMC),^[^ ^6^, ^10^ ^]^ but most cells in PBMC (especially macrophages and monocytes) are lost during culture, leading to inaccurate prediction of patient's drug responses.^[^ ^6b^ ^]^

MSC are multi‐potent cells that can be easily isolated from bone marrow, umbilical cord blood and adipose tissues.^[^ ^11^ ^]^ Due to their great plasticity and non‐immunogenic property, allogenic MSC can be expanded in vitro and used in the clinical treatment of autoimmune and degenerative diseases.^[^ ^12^ ^]^ Studies have demonstrated that bone‐marrow‐derived MSC (BM‐MSC) can be recruited to the tumor site and differentiate into CAF,^[^ ^11^, ^13^ ^]^ but whether they can support TME formation during in vitro PDO culture remains unknown. In addition, the PDOs cultured on cell culture plates display heterogeneous sizes and cellular composition,^[^ ^14^ ^]^ therefore, are unsuitable for high‐throughput drug screening. In this regard, microfluidic chips consist of microarrays with various geometries which can be designed for 3D culture and tailored to mimic controlled sizes and drug delivery.^[^ ^15^ ^]^ Nowadays, microfluidics technology have been applied in organoid engineering and research, providing an ideal and inexpensive platform for high‐throughput PDO culture and drug screening.^[^ ^16^ ^]^

In the present study, we established an MSC‐PDO‐PBMC co‐culture system to enhance the success rate and growth rate of HCC organoid culture and mimic the TME by supporting monocyte/macrophage survival and TAM differentiation. In addition, we designed a microfluidic chip to improve the homogeneity of high‐throughput MSC‐PDO‐PBMC co‐culture and achieve dynamic drug delivery. This microengineered organoid‐on‐a‐chip using designed microwells and channels to improve tumor engineering models mimicking TME not only dramatically reduced the time and cost of HCC organoid culture and high‐throughput drug screening but also showed more precise prediction potential in assessing HCC patients’ immunotherapeutic response, therefore provides a high‐efficient platform for personalized drug screening.

## 2 Results

### 2.1 Design of HCC Organoid‐on‐a‐Chip for High‐Throughput Drug Screen

**Figure 1:**
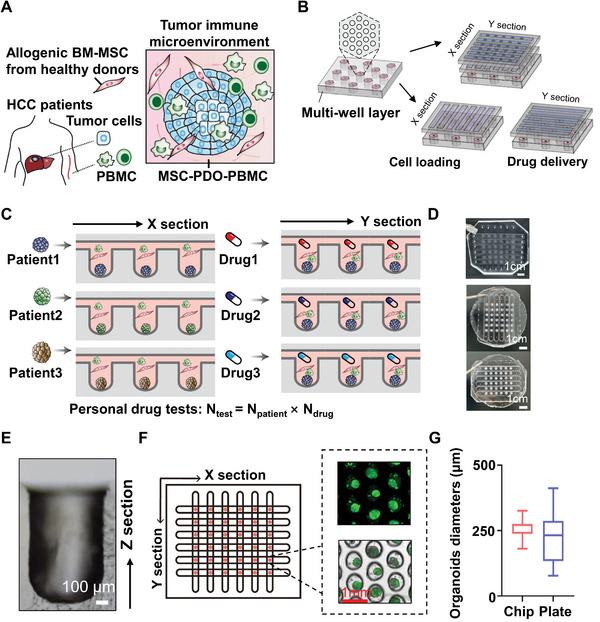
PDO from HCC specimens cocultured with MSC and PBMC to establish HCC TME on high‐throughput microfluidic chips. A) PDO was obtained from primary HCC specimens, cocultured with autologous PBMC and allogenic bone marrow‐derived MSC from healthy donors to establish TME. B) Scheme of a microfluidic chip for high‐throughput organoid culture and drug screen. The top and middle layer of microchips were respectively designed for drug delivery (Y section) and cell loading (X section), and each layer contains 6 microchannels that are vertically cross‐distributed; while the bottom layer for organoid culture and drug test contains 36 microarray units, each with 19 microwells arrayed in a hexagon. The scheme showed two encapsulation ways. C) Workflow of high‐throughput microchips for PDO culture and drug tests. D) Photographs of the fabricated chips with two encapsulation ways: three‐layer (top) or two‐layer separately (middle and bottom). E) cross‐sectional view of the microwell was shown. F) Each of the 36 microarray units was located at the X (cell loading) and Y (drug delivery) intersection, and representative bright‐field and fluorescence‐staining images of PDOs cultured on microarray chip were shown. G) Comparison of dimensional variations of HCC organoids cultured on microarray chips or 96‐well plates after 7‐day culture. Size differences of the PDOs cultured on microarray chips versus 96‐well plates were calculated, and the diameter derivation was shown (*n* = 19). PDO, patient‐derived organoid; HCC, Hepatocellular carcinoma; MSC, Mesenchymal stromal cells; PBMC, peripheral blood mononuclear cells.

For immunotherapeutic drug screen, HCC tumor cells obtained from needle biopsies or surgical resections were co‐cultured with autologous PBMC and allogenic bone marrow derived MSC (BM‐MSC) from healthy donors to reconstruct the TME (**Figure** **1**A). We also designed a microfluidic chip for high‐throughput organoid culture and drug screen and the scheme was shown in Figure 1B. The top and middle layer of microchips were respectively designed for drug delivery (Y section) and cell loading (X section), and each layer contains 6 microchannels that are vertically cross‐distributed; while the bottom layer for organoid culture and drug test contains 36 microarray units, each with 19 microwells arrayed in a hexagon. Our chip has two encapsulation ways, and the details are shown in the experimental section (Figure 1B; Figure S1 and Movie S1, Supporting Information).

As shown in Figure 1C, mixed MSC‐PBMC‐PDO can be loaded through the middle layer and arrested into the bottom layer microwells by turbulent flow stress, cultured to form HCC organoid mimicking TME, followed with dynamic drug delivery from top layer channels. We can use this microchip to achieve high‐throughput PDO culture and personal drug tests. Photographs of two fabricated chips and a cross‐sectional view of the microwell were shown (Figure 1D,E). Each microwell is 600 µm in diameter and 900 µm in depth, with a U‐shape bottom to increase cell‐cell interaction to benefit tumor spheroid formation, and 19 microwells form a microarray unit for drug testing.

We first optimized the cell loading conditions and found that when cell suspensions were carefully pumped into the microchannels with an optimal velocity of 10 µL min^−1^, at an optimal density of 5×10^4^ mL^−1^, PDO can be equally distributed into all microarray units and quickly formed organoids with high uniformity (Figure S2A,B, Supporting Information). Representative bright‐field and fluorescence‐staining images of PDO cultured on a microarray chip were shown (Figure 1F; Figure S2C, Supporting Information), showing the distribution of PDOs on the chip. HCC organoids were cultured on microchips for 7 days and then observed by microscopy, and the dimensional variation of PDOs (as indicated by the dispersion of PDO diameters) cultured in microchips was much lower than that of conventional culture on 96‐well plates (Figure 1G). Most cocultured PDOs displayed similar cell composition pattern, for each cell population could be found in the PDOs (Figure S2D, Supporting Information), suggesting that our microchips dramatically improved the uniformity of high‐throughput PDO culture.

### 2.2 MSC Promote PDO Culture from HCC Biopsy or Resection Specimens

**Figure 2:**
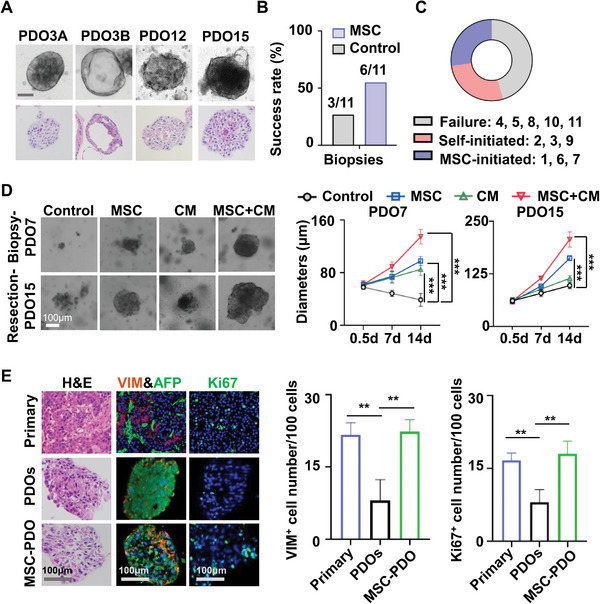
MSC support PDO culture from HCC biopsy or resection specimens. A) HCC PDOs with distinct morphology, as assessed by bright‐field microscopy (up) or H&E staining (down). B) Success rates of PDO culture from HCC biopsy samples with/without MSC. C) The pie chart shows the PDO culture results of 11 HCC biopsy samples. D) PDOs were cultured with or without MSC and/or 30%(v/v) MSC‐CM, and their growth was recorded using microscopy. Representative images and growth curves of PDOs derived from biopsy (PDO7) or resection (PDO15) were shown (*n* = 5). E) H&E and immunofluorescence staining of primary HCC specimens and their organoids cocultured with or without MSC for 7 d. AFP (green, liver cancer marker), Vimentin (red, stromal cell marker), Ki67 (green, cell proliferation marker), and DAPI (nuclei staining). The number of Vimentin‐ and Ki67‐positive cells were examined by microscopy and calculated per 100 cells. Data were shown as mean ± SD (*n* = 3). *p*‐values were calculated with two‐tailed unpaired *t*‐test. **, *p*<0.01; ***, *p*<0.001. CM, conditioned culture medium; PDO, patient‐derived organoid; HCC, Hepatocellular carcinoma; MSC, Mesenchymal stromal cells; PBMC, peripheral blood mononuclear cells.

Next, we investigated the effects of MSC on PDO culture from primary HCC specimens. 11 biopsy and 11 resection samples were used. HCC PDO cultures were successfully established in 12 specimens (3 biopsies and 9 resections), whereas others failed. Most PDOs presented compacted spheroid‐like structures (solid type), while others grew as single‐layered epithelium surrounding a central lumen (luminal type) (**Figure** **2**A). Among 12 established PDO cultures, 7 PDOs (PDO3, 15, 16, 18, 19, 20, 22) were able to continue growing after passaging and cryopreservation, while others (PDO2, 9, 12, 14, 17) gradually stopped expansion (Figure S3A, Supporting Information). The success rate of PDO cultures from biopsies and surgery resection samples were, respectively 3 out of 11 (27%) and 9 out of 11 (82%) (Figure S3B, Supporting Information). In comparison, MSC improved the success rate of biopsy‐derived PDO culture from 27% (3 out of 11, self‐initiated) to 54% (6 out of 11, including 3 self‐initiated and 3 MSC‐initiated) (Figure 2B,C), but did not change the success rate using resection samples (82%, 9 out of 11, all self‐initiated) (Figure S3C,D, Supporting Information).

We also found that co‐culture with MSC dramatically accelerated, while supplement with MSC's conditioned medium (CM) slightly promoted organoid growth in PDOs from both biopsy and resection samples, and the promotive effects from high to low were: MSC+CM > MSC > CM > control. Representative images and growth curves of PDO7 (biopsy‐derived and MSC‐initiated, solid type) and PDO15 (resection‐derived and self‐initiated, solid type) were shown at day14 after MSC co‐culture or CM treatment (Figure 2D), confirming the general promotive effect of MSC on PDO growth. To be noted, we found that CM could promote tumor organoid growth even in a basic culture medium without supplement of indicated cytokines (Figure S4, Supporting Information). Furthermore, hematoxylin and eosin (H&E) and immunofluorescence staining showed that original HCC tissues expressed alpha‐fetoprotein (AFP, liver cancer marker) and vimentin (VIM, stromal cell marker) and exhibited high expression of Ki67 (cell proliferation marker). These features were observed in PDOs co‐cultured with MSC rather than conventional cultured PDOs (Figure 2E).

### 2.3 MSC and CAF Show Similar Abilities in Promoting PDO Growth

**Figure 3:**
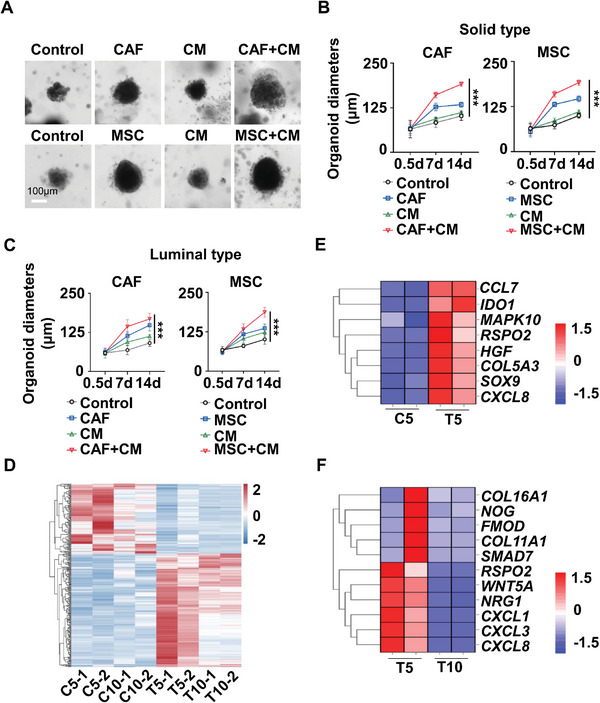
MSC and CAF show similar abilities in promoting PDO growth. A–C) PDOs with a typical solid sphere or luminal shape were cultured with or without autologous cancer‐associated fibroblast (CAF) or allogenic MSC in the presence or absence of their respective CM (30% v/v), and the organoid growth was recorded using microscopy. A) Representative images of solid‐type PDOs at day 14 after culture were shown. Growth curves of the solid sphere‐ (B) or luminal‐shaped (C) PDOs were shown (*n* = 5). D) RNA‐seq analysis of transcriptome profile in MSC at Passage 5 or 10 before (control‐treated MSC, C5/10) and after treatment with PDO‐conditioned medium (tumor‐educated MSC, T5/10) were shown in the heatmap. (E&F) Gene expression profiles were compared in MSC (C) versus TA‐MSC (T) or in TA‐MSC at Passage 5 (T5) versus Passage 10 (T10), as shown in the heatmap. Data were shown as mean ± SD. *p*‐values were calculated with two‐tailed unpaired *t*‐test. ***, *p*<0.001. CM, conditioned culture medium; PDO, patient‐derived organoid; MSC, Mesenchymal stromal cells; CAF, Cancer‐associated cells.

We also isolated CAF from HCC resection specimens, which showed similar morphology and featured phenotype as BM‐MSC (Figure S5A,B, Supporting Information). Both allogenic MSC and autologous CAF showed comparable effects on supporting HCC PDO culture organoid growth when co‐cultured with PDO15 (solid type) or PDO3 (luminal type). And the promotive abilities from high to low were: MSC+CM > MSC > CM > control or CAF+CM > CAF > CM > control (**Figure** **3**A–C).

To assess whether MSC can differentiate into CAF in the HCC microenvironment and whether passage number affects MSC's plasticity, we used MSC at different passages to co‐culture with PDOs and found that MSC under Passage 10 (P10) displayed more potent abilities to promote PDO growth than those with higher passages (Figure S6, Supporting Information). In this regard, MSC at P5 or P10 were treated with PDO supernatant to generate tumor‐educated MSC (TA‐MSC), and the gene expression profile of TA‐MSC versus untreated MSC was assessed by RNA‐seq. MSC at P5 but not P10 showed strong response to tumor education (Figure 3D–F), as indicated by the upregulated genes involved in tumor growth, chemoattractant, and immune suppression. We further confirmed the gene expression pattern in CAF versus MSC at P5 or P10 by real‐time polymerase chain reaction (PCR) (Figure S7, Supporting Information), and found that MSC at P5 but not P10 displayed a CAF‐like phenotype after tumor education, indicating that MSC gradually lost its ability of differentiation after 10 passages. Therefore, we used MSC at P5‐8 in the following experiments to obtain optimal effects.

### 2.4 MSC Promote Monocyte/Macrophage Survival and M2 Differentiation

**Figure 4:**
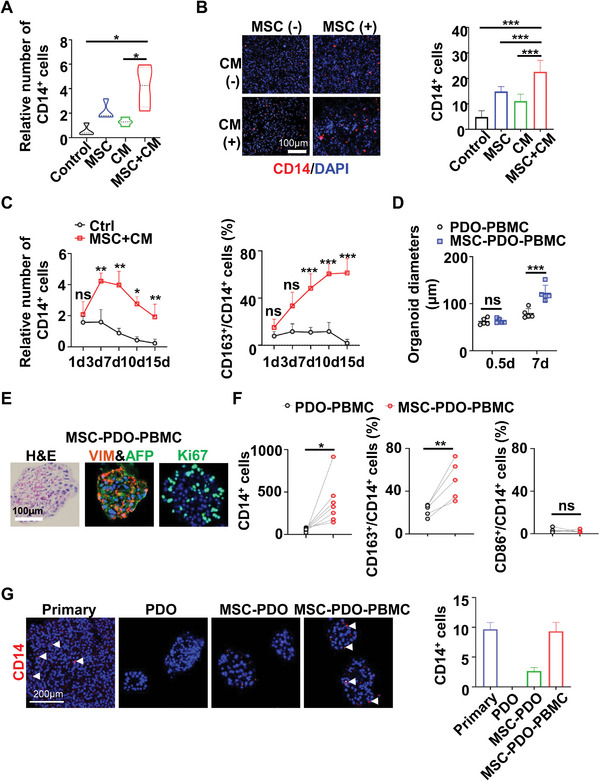
MSC promote monocyte proliferation and M2 differentiation to reconstruct TME. A,B) PBMC were cultured with or without MSC (MSC: PBMC = 1:30) and/or their CM (30%v/v) for 7 days. CD14‐positive monocytes were examined by A) flow cytometry (*n* = 4) or B) immunofluorescence staining (*n* = 5). C) The increased fold of CD14^+^ monocytes (*n* = 6) and CD163^+^ M2 cells (*n* = 7) were assessed by flow cytometry in PBMC cultured with or without MSC plus CM. D) HCC‐PDO and their autologous PBMC were cocultured with or without allogenic MSC at appropriate ratios. Organoid diameters were assessed by microscopy (*n* = 5). E) H&E and immunofluorescence staining of AFP (green), Vimentin (red), Ki67 (green), and DAPI (blue) in MSC‐PDO‐PBMC cultured for 7 d. Scale bar 100 µm. F) Flow cytometry analysis of CD14‐positive monocytes (*n* = 7) and M1/M2 differentiation (*n* = 5) in PDO‐PBMC versus MSC‐PDO‐PBMC. G) CD14^+^ monocytes/macrophages (red, white arrows) in HCC primary tissues and their corresponding PDO coculture models were examined by immunofluorescent microscopy and calculated per 100 cells. Scale bar 200 µm. Data are shown as mean ± SD (*n* = 3). *p*‐values were calculated with two‐tailed unpaired *t*‐test. ns, not significant, *, *p* < 0.05; **, *p*<0.01; ***, *p*<0.001. CM, conditioned culture medium; PDO, patient‐derived organoid; HCC, Hepatocellular carcinoma; MSC, Mesenchymal stromal cells; PBMC, peripheral blood mononuclear cells.

As the dominant cell type in the tumor immune microenvironment, monocytes/macrophages are easily lost during PDO culture, while stromal cells are crucial in regulating monocyte/macrophage survival and differentiation.^[^ ^17^ ^]^ To investigate the role of MSC in the long‐term in vitro culture of monocytes/macrophages, PBMC were co‐cultured with MSC at different ratios or treated with MSC‐derived CM for more than 7 days and then analyzed by flow cytometry or immunostaining. MSC or CM enhanced the monocyte number in a ratio‐ or dose‐dependent manner, and co‐culture of MSC with PBMC at 1:30 or addition with 30%(v/v) CM was optimal (Figure S8A,B, Supporting Information). Moreover, MSC and CM showed a synergistic effect in promoting monocyte/macrophage survival (**Figure** **4**A,B).

To be noted, MSC+CM dramatically promoted monocyte proliferation within 7 days and maintained their survival until 15 days, whereas the Macrophage colony‐stimulating factor (M‐CSF) supplement (control group) slightly promoted monocyte proliferation within 3 days but could not sustain monocyte survival after 3‐day culture (Figure 4C). MSC+CM treatment enhanced monocyte differentiation toward CD163^+^ M2 but not CD86^+^ M1 macrophages (Figure 4C; Figure S8C,D, Supporting Information), and slightly decreased CD3^+^ T lymphocyte number upon Interleukin (IL)‐2 stimulation (Figure S8E, Supporting Information). PCR and Western blot data further confirmed that MSC promotes M2 differentiation, as indicated by the upregulated CD206, IL10 and Arginase‐1 expression in MSC‐treated monocytes (Figure S9, Supporting Information). These data indicated that MSC favors macrophage differentiation toward TAM‐like (M2) phenotype.

### 2.5 MSC‐PDO‐PBMC Co‐Culture Generates Organoids Similar to the Original TME

To generate a PDO model mimicking TME in the primary tumor, HCC PDOs and their autologous PBMC were co‐cultured with MSC at an optimal ratio of MSC:PDO: PBMC (1:10:30). Consistent with the observation in MSC‐PDO co‐culture system, MSC promoted PDO growth in the presence of autologous PBMC (Figure 4D; Figure S10A, Supporting Information). Immunostaining data showed that HCC organoids generated by MSC‐PDO‐PBMC models highly expressed AFP, VIM, and Ki67 (Figure 4E). These features were detected in primary HCC tissues but absent in conventional cultured PDO (Figure 2E). Moreover, flow cytometry analysis showed that MSC dramatically promoted monocyte survival and M2 differentiation in PDO‐PBMC co‐culture, but did not affect M1 polarization (Figure 4F). CD14+ macrophages were detected in MSC co‐cultured organoids by immunostaining, while organoids cultured with MSC plus PBMC showed a large number of macrophages as in primary HCC tissues (Figure 4G), indicating that MSC‐PDO‐PBMC co‐culture generated organoids similar to original HCC TME.

We also compared the role of allogenic MSC versus autologous CAF in organoid growth and TME formation, and found that both showed similar effects on promoting PDO growth, macrophage survival, and M2 differentiation (Figure S10B–D, Supporting Information).

### 2.6 MSC‐PDO‐PBMC and Conventional PDO Models Show Comparable Predictions of Chemotherapeutic or Targeted Drug Response

To evaluate which model is more suitable for personalized drug screening, MSC‐PDO‐PBMC and CAF‐PDO‐PBMC versus conventionally cultured PDO models were treated with Sorafenib, Oxaliplatin, 5‐Fluorouracil (5‐FU), and Cisplatin. At 6 days post‐treatment, cell viability was assessed by Cell Counting Kit‐8 (CCK‐8), and dose‐response curves were plotted to determine the half‐maximal inhibitory concentrations (IC50). We found that PDO3 and PDO15 showed different drug responses to Sorafenib, but similar drug responses to Oxaliplatin, 5‐FU, and Cisplatin, while co‐culture with allogenic MSC or autologous CAF did not change the PDOs’ sensitivity to these chemotherapeutic or targeted drugs (Figures S11 and S12A, Supporting Information).

**Figure 5:**
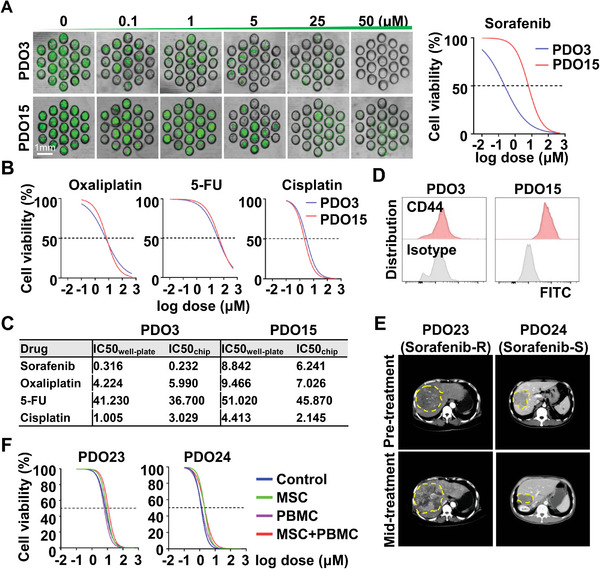
Chemotherapeutic agents sensitivity tests of MSC‐PDO‐PBMC models. A) Drug sensitivity test on microfluidic chips using MSC‐PDO‐PBMC models. Calcein AM‐prestained PDO3, and PDO15 were cocultured with MSC and PBMC and then treated with Sorafenib at indicated doses. Representative microscopy images were shown 6 days after Sorafenib treatment and dose‐response curves were shown. B) Dose‐response curves of PDO3 and PDO15 based on the MSC‐PDO‐PBMC model to Oxaliplatin, 5‐FU, and Cisplatin, as tested on microchips. C) Comparison of IC50 of PDO3 and PDO15 coculture models to chemotherapeutic drugs as performed on 96‐well‐plate versus microchips. D) CD44 was assessed by flow cytometry in PDO3 and PDO15. E) PDO23 and PDO24 were respectively isolated from Sorafenib‐resistant and sensitive HCC patients, and CT scan images of their corresponding patients before and after 3‐month treatment with Sorafenib were shown. F) PDO23 and PDO24 cocultured with/without MSC and/or PBMC were treated with Sorafenib at different doses, and their dose‐response curves were shown. CT, computed tomography; PDO, patient‐derived organoid; MSC, Mesenchymal stromal cells; PBMC, peripheral blood mononuclear cells.

Next, we performed drug sensitivity tests of PDO3/PDO15 on microchips based on immunofluorescence microscopy, and analyzed the organoid mean area per microarray unit by Image J to reflect the cell viability. Data showed that PDO3 was more sensitive to Sorafenib than PDO15 (**Figure** **5**A) and similar drug sensitivity to Oxaliplatin, 5‐FU and Cisplatin (Figure 5B). Data showed that IC50 of PDO3/PDO15 based on MSC‐PDO‐PBMC co‐culture on microchips were similar to that in 96‐well plates (Figure 5C), but the time and cost of drug sensitivity test using organoid‐on‐a‐chip are much less than conventional methods. Studies have identified CD44 as an indicator of Sorafenib resistance.^[^ ^18^ ^]^ We found that PDO15 positively expressed CD44 and PDO3 were CD44‐negative (Figure 5D), consistent with their IC50 results.

To further evaluate the prediction accuracy of the PDO‐based drug sensitivity test, we used previously cryopreserved biopsy‐derived PDOs from HCC patients with different Sorafenib responses (PDO23: Sorafenib‐resistant, PDO24: Sorafenib‐sensitive, as indicated by computed tomography (CT) images before and after Sorafenib treatment in Figure 5E), to assess their drug sensitivity based on the co‐culture system with or without MSC/PBMC, and the results were consistent with their corresponding patients’ responses (Figure 5F), indicating the great potential for clinical application of the drug‐screen microchips.

### 2.7 MSC‐PDO‐PBMC Model Predicts Immunotherapeutic Drug Response More Accurately than Conventional PDOs

Clinically, only 20% of HCC patients benefit from anti‐PD‐1/PD‐L1 immunotherapy. Current predictions of suitable patients for anti‐PD‐1/PD‐L1 treatment are mainly dependent on high PD‐L1 expression, high tumor mutation burden (TMB‐H and MSI‐H/dMMR), and immune cell infiltration,^[^ ^3^, ^19^ ^]^ but an in vitro drug response prediction model remains lacking.

**Figure 6:**
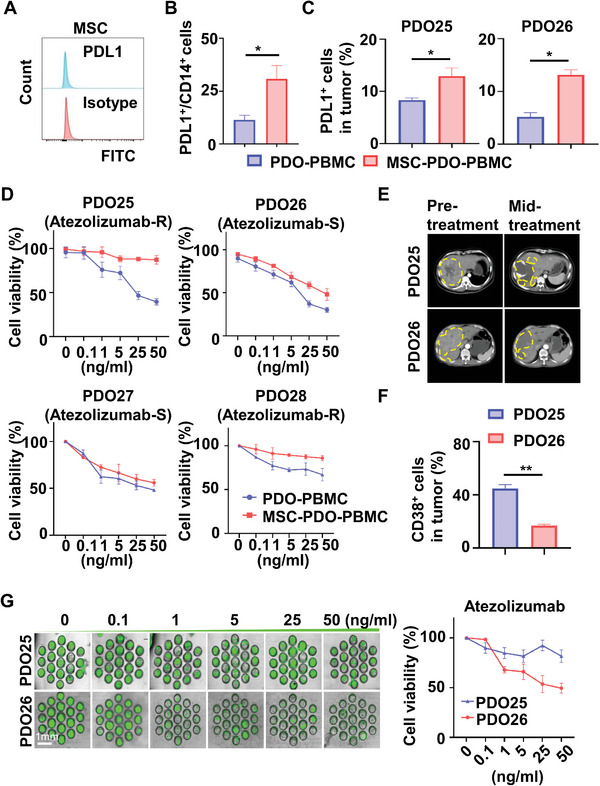
MSC‐PDO‐PBMC model predicts immunotherapeutic drug responses. A,B) Flow cytometry analysis of PD‐L1 protein levels on in vitro expanded MSC (A) or monocytes/macrophages in coculture models (*n* = 3) (B). C) PD‐L1 expression on tumor cells of PDO25 and PDO26 coculture models was detected by flow cytometry (*n* = 3). D) PDO25‐28 coculture models were treated with Atezolizumab at different doses for 6 days. Cell viabilities of tumor cells in the MSC‐PDO‐PBMC versus PDO‐PBMC model were detected by flow cytometry with Annexin V‐FITC staining (*n* = 2). E) PDO25 and PDO26 were respectively isolated from Atezolizumab‐resistant and sensitive HCC patients, and CT scan images of their corresponding patients before and after 3‐month Atezolizumab (anti‐PD‐L1) therapy were shown. F) CD38 expression on tumor cells in each coculture model was examined by flow cytometry (*n* = 3). G) Immunotherapy drug sensitivity tests were performed on microfluidic chips using PDO25/26 coculture models (*n* = 2). Representative microscopy images at 6 days were shown, and cell viability was calculated. Scale bar = 1 mm. Data were shown as mean ± SD. *p*‐values were calculated with two‐tailed unpaired *t*‐test. *, *p*<0.05; **, *p*<0.01. CT, computed tomography; PDO, patient‐derived organoid; MSC, Mesenchymal stromal cells; PBMC, peripheral blood mononuclear cells.

Here, we evaluated the immunotherapeutic drug sensitivity of PDO co‐culture models. We found that normally expanded MSC did not express PD‐L1 (**Figure** **6**A) but enhanced PD‐L1 expression on monocytes/macrophages and tumor cells during co‐culture (Figure 6B,C). We also used PDOs from HCC patients with different responses to Atezolizumab (anti‐PD‐L1 Ab), and constructed the MSC‐PDO‐PBMC model to assess the sensitivity of immunotherapy (Figure 6D). Based on clinical information, the patient of PDO25, 28 presented progressive disease (PD), while the patient of PDO26, 27 presented partial remission (PR) after Atezolizumab treatment (Figure 6E; Figure S13, Supporting Information). High CD38 expression in tumors is often associated with resistance to anti‐PD‐1/PD‐L1 immunotherapy.^[^ ^20^ ^]^ Our flow cytometry data showed that PDO25 displayed much higher expression of CD38 than PDO26 (Figure 6F), while their PD‐L1 expression was comparable, which is consistent with their clinical response to Atezolizumab.

We utilized different co‐culture models to test the drug sensitivity of PDO25 and PDO26 to Atezolizumab on 96‐well plates. For PDO25 (Atezolizumab‐resistant), both MSC‐PDO‐PBMC and CAF‐PDO‐PBMC models showed a resistant phenotype to Atezolizumab, while the PDO‐PBMC model displayed a sensitive drug response. Whereas for PDO26 (Atezolizumab‐sensitive), MSC‐PDO‐PBMC, CAF‐PDO‐PBMC, and PDO‐PBMC models showed a sensitive response to Atezolizumab (Figure 6D; Figure S12B, Supporting Information). These data together indicate that MSC(CAF)‐PDO‐PBMC models mimicking original TME were more suitable than conventional PDO models for in vitro tests of immunotherapeutic drugs.

Finally, we performed the drug sensitivity test on microchips based on MSC‐PDO‐PBMC co‐culture and found that the results were consistent with the CCK‐8 assay in 96‐well plates (Figure 6G). This microengineered organoid‐on‐a‐chip mimicking TME shortened the organoid culture time within 1 week, providing an efficient and high‐throughput platform for immunotherapeutic drug screening.

## 3 Discussion

PDO‐based personalized drug screening has great potential for precision oncology, but some drawbacks of current PDO culture techniques, such as low success rate, time‐consuming and lacking of TME, limit their clinical application. In the present study, we established the MSC‐PDO‐PBMC co‐culture system to promote HCC PDO growth and TME formation, as well as designed a microfluidic chip platform for high‐throughput organoid culture and drug screening.

Clinically, since HCC progresses rapidly and lacks efficient therapies or drugs, treatment decisions need to be made as soon as possible. However, current methods often take one month for HCC PDOs to grow up to ≈200 µm in diameter for drug screening, leading to unacceptable treatment delays. Moreover, the success rate of HCC biopsy‐derived PDO culture is less than 30%,^[^ ^9^ ^]^ limiting their clinical application for precision oncology. Tumor stromal cells have been reported to accelerate tumor growth and tumor sphere formation.^[^ ^21^ ^]^ Thus, we used autologous CAF or allogenic BM‐MSC to support PDO culture and found that both CAF and MSC dramatically promoted PDO growth and shortened the culture time to 1–2 weeks. MSC also increased the success rate of HCC PDO culture from biopsies by two times, further improving the clinical application possibility of PDO‐based personalized drug screens.

Immunotherapy using ICI inhibitors is a novel and promising treatment for HCC, but only less than 20% of HCC patients benefit from this treatment. Some studies have tried to use the PDO model to predict patients’ drug responses, but it is hard to evaluate their immunotherapy response based on conventional PDO culture due to lacking the microenvironment, especially the immune cells. TAM are the most abundant TME cells with M2‐like phenotype.^[^ ^4b^ ^]^ Monocyte‐derived macrophages from PBMC are recruited into TME by stromal cells, then differentiate into TAM, but monocytes/macrophages can hardly survive during long‐period in vitro culture. Studies have proved that immune populations are easily lost in PDOs.^[^ ^6b^ ^]^ Our experimental data also showed that PDO cocultured with primary immune cells faced the problem of PBMC loss. In Figure 4, immune cells were hardly detected in PDO and PDO‐PBMC coculture models after 7d culture, indicating that PBMCs were quickly lost without MSC coculture. In TME, the second most abundant immune cells are T cells, and most tumor‐infiltrating T cells are bystander T cells, while other tumor‐specific cytotoxic T cell activities are inhibited in immune‐suppressive TME. Stromal cells play an essential role in tumor growth and TME formation. Thus, HCC patient‐derived PDO and PBMC were co‐cultured with either autologous CAF or allogenic BM‐MSC to reconstruct TME in vitro.

Our data showed that BM‐MSC displayed a similar phenotype as CAF, and both MSC and CAF displayed similar promotive effects on HCC organoid growth, macrophage survival, and TAM differentiation via cell‐cell interaction and paracrine factors. CAF are terminally differentiated cells, and isolation of CAF is time‐consuming, with a low success rate, limiting their clinical application. While MSC are multi‐potent cells with great plasticity, non‐immunogenic property, and wide sources. Studies have identified MSC as a major precursor of tumor stromal cells in distinct tumor types^[^ ^11^, ^13^ ^]^ and demonstrated that MSC could differentiate into many kinds of CAF, as detected by expression of alpha‐smooth muscle actin, fibroblast surface protein, and persistence of tumor‐supportive ability. Our results also found that MSC could become a CAF‐like phenotype to promote PDO growth and construct an immunosuppressive microenvironment (Figure S7, Supporting Information). MSC‐P5 showed similar gene expression pattern with CAF after tumor CM education, indicating that our MSC‐PDO‐PBMC model has an excellent potential for extension to a wide variety of other tumors. Thus, compared to CAF, MSC is more suitable for organoid culture and personalized drug screening. To be noted, MSC's abilities to maintain TME gradually reduced after multiple cell passages. In the present study, BM‐MSC at P5 showed more potent plasticity than P10, upon stimulation with HCC PDO‐conditioned media, MSC‐P5 displayed a similar gene profile as primary HCC‐CAF, while P10 cells lost their differentiation abilities. Therefore, we used allogenic BM‐MSC at P5‐P8 in the MSC‐PBMC‐PDO coculture system to reconstruct HCC TME in vitro to establish a novel efficient drug screen platform.

To be noted, PDO‐PBMC co‐culture is often used as an organoid‐killing model, in which IL‐2 is added to activate tumor‐reactive T cells. It is very different from the original suppressive TME and, therefore, cannot be used as in vitro evaluation model for immunotherapy. While in our model, in the presence of IL‐2 (2000IU/ml), MSC/CAF plus CM slightly suppressed T cell proliferation, thereby allowing the establishment of HCC organoids mimicking the immune suppressive TME.

Conventional PDOs cultured on plates often display varying sizes and cellular compositions.^[^ ^22^ ^]^ Microfluidic chips consist of microarray units designed for 3D cell culture and tailored to mimic controlled dimensions and drug delivery. This provides an ideal, inexpensive platform for high‐throughput PDO culture and drug screening.^[^ ^23^ ^]^ In the present study, we designed a multi‐layer microfluidic chip to improve the uniformity of high‐throughput cultured PDOs. Since the volume of each microwell is approximately one‐thousandth of a culture well on a 96‐well plate, the cost and time frame of PDO culture and drug screening can be reduced to a great extent. Moreover, the microchannels on the top layer also provided a dynamic drug delivery mode to mimic in vivo drug administration, thereby improving drug test accuracy.^[^ ^24^ ^]^

Finally, we used the MSC‐PDO‐PBMC model to examine the HCC organoids’ responses to common chemotherapeutic, molecular targeted, and immunotherapeutic drugs for HCC, including Oxaliplatin, 5‐FU, Cisplatin, Sorafenib, and Atezolizumab. Since most of the HCC patients enrolled in the present study did not receive targeted drug treatment or immunotherapy after biopsy or surgical operation, we verified the accuracy of the MSC‐PDO‐PBMC model using previously cryopreserved biopsy‐derived PDOs from HCC patients with different drug responses to subsequent Sorafenib or Atezolizumab treatment. Compared to the conventional cultured PDO model, the MSC‐PDO‐PBMC model provides more precise predictions of HCC patients’ response to the immunotherapeutic drug Atezolizumab due to the reconstruction of tumor immune microenvironment in our co‐culture system. We further utilized microarray chips to achieve high‐throughput MSC‐PDO‐PBMC co‐culture and drug screening and found that the drug sensitivity assessed by fluorescence microscopy on microchips was similar to that determined by CCK‐8 assay on plates, but the time frame of organoid culture was reduced to one week only.

In the present study, we successfully constructed a microengineered HCC organoid‐on‐a‐chip based on the MSC‐PDO‐PBMC co‐culture model to simulate HCC TME and enhance the efficacy and uniformity of organoid culture. More importantly, this microfluidic platform dramatically reduced the time frame for high‐throughput organoid culture and drug screening and displayed more accurate predictions of clinical HCC patients’ responses to common anti‐tumor drugs, especially the immunotherapeutic drugs like ICI, therefore exhibiting a great potential in personalized cancer therapy and immunotherapy prognosis predictions.

## 4 Experimental Section

### Clinical Sample Collection and Ethical Statements

Tumor specimens from 17 needle biopsies or 11 surgical resections were obtained from 28 HCC patients in the First Affiliated Hospital of Sun Yat‐sen University from 2020 to 2022. The patients’ clinical information is shown in Table S1 (Supporting Information). MSC were isolated from healthy donor bone marrow and CAF from the HCC resection sample, using the adherent culture method.^[^ ^2^ ^5]^ PBMCs were isolated from HCC patients or healthy donors using Ficoll density gradient centrifugation. The study was approved with the patient's informed consent and ethical approval (FAH‐SYSU‐2018[43] and ZSSOM‐2021[077]).

### Culture of HCC PDOs

HCC PDO culture was performed as in the previous study.^[^ ^7^, ^26^ ^]^ Samples were minced and digested with 2 mg mL^−1^ collagenase II (GIBCO) at 37 °C for 1 h. Then, pellets were filtered through a 70‐µm cell strainer (Falcon), mixed with Matrigel (Corning), and cultured in HCC organoid culture medium: Advanced DMEM/F12 medium (GIBCO) was supplemented with 1% Penicillin/Streptomycin (Biological Industries), 1% L‐Glutamine (STEMCELL), 10 mm HEPES (GIBCO), 1:50 B27 supplement (without Vitamin A, GIBCO), 1:100 N2 supplement (GIBCO), 1.25 mm n‐Acetyl‐L‐cysteine (Sigma), 10 mm nicotinamide (Sigma), 50 ng mL^−1^ recombinant human R‐spondin (Rspo)‐1 (Novoprotein), 50 ng mL^−1^ recombinant human Epidermal growth factor (EGF) (Novoprotein), 50 ng mL^−1^ recombinant human Fibroblast growth factor (FGF) 10 (Novoprotein), 50 ng mL^−1^ recombinant human Hepatocyte growth factor (HGF) (Novoprotein), 10 ng mL^−1^ Noggin (Novoprotein), 5 µm A8301 (Abmole) and 10 µm Y27632 (Abmole). Organoids were digested using collagenase II, washed, and cultured in HCC organoid culture medium with Matrigel for subsequent culture.

### Co‐Culture of MSC‐PDO‐PBMC

For MSC‐PBMC coculture, PBMCs were incubated with CM at the proportion of 10% to 50%, and/or cultured with MSC at different ratios (MSC: PBMC = 1:5 to 1:100) in 96‐well plates in RPMI1640 complete medium with 20 ng mL^−1^ recombinant human M‐CSF (PeproTech) for more than 7 days. To construct MSC‐PDO co‐culture models, after mincing and digestion, primary tumor cells derived from HCC specimens were mixed with MSC at a ratio of 10:1 and then cultured in HCC organoid culture medium containing Matrigel and 30%(v/v) conditioned medium (CM) for more than two weeks for further analysis. To establish the MSC‐PDO‐PBMC co‐culture system, tumor cells derived from HCC specimens were mixed with autologous PBMC and allogenic MSC at a ratio of 10:30:1, and then cultured in HCC organoid culture medium containing Matrigel and 30%(v/v) CM plus 20 ng mL^−1^ M‐CSF for more than 7 days.

### Design and Fabrication of Microfluidic Chips

Microfluidic chips were produced with the following protocol described previously.^[^ ^15^, ^27^ ^]^ Briefly, the devices were designed using Fusion360 software (Autodesk Inc. CA, USA), and the corresponding resin molds were printed in SLA 3D printer (Asiga PICO2 HD 27). Microfluidic chips were produced by standard soft lithography using polydimethylsiloxane (PDMS, SYLGARD 184 Silicone Elastomer Kit, Dow Corning). The microfluidic chip was composed of three layers. All the layers were fabricated by casting the pre‐polymer of PDMS part A and part B (10:1) onto a mold and curing at 80 °C for 1 h. There were two encapsulation ways of the chip. The first way was to encapsulate with three layers. There were hollow channels (with a diameter of 2 mm) in the middle layer, connecting the top layer and the bottom‐layer microwells. When introducing fluids into one layer, all the inlets and outlets of the other layer were plugged up, allowing the drugs to be loaded from the top layer into the bottom‐layer wells via those hollow channels. The second way was to bond the middle or top layer to the bottom layer respectively, to separate the progress of cell loading and drug delivery. The middle layer was removed and the top layer was bonded to the bottom layer when drug delivery. Before loading cells, devices were sterilized with 75% (v/v) alcohol and washed with PBS. Organoids were added to the microarray chip through the middle layer. Drugs with indicated doses were added through the top layer.

### Drug Sensitivity Tests

Tumor organoids were treated with Sorafenib, Cisplatin, Oxaliplatin and 5‐FU (MedChemExpress), and Atezolizumab (Roche) at indicated concentrations. PDO mixed with a culture medium containing 10% Matrigel was pumped into microchips using microsyringes at a controlled flow velocity (10 µL min^−1^). After organoids reached around 200 µm diameter in size, drugs were carefully delivered into microchips using microsyringes, followed by another 6‐day culture. Organoids cultured on microchips were visualized by microscopy. For drug screening on 96‐well plates, cell viability was assessed by CCK‐8 assay (Glpbio, GK10001) or Annexin V‐FITC (ES Science) staining with flow cytometry, according to the manufacturer's instruction. Dose‐response curves were analyzed by GraphPad Prism software (Dotmatics), and the mean area of total PDOs in each microarray unit was analyzed by Image J software.

### Histology and Immunofluorescence

Tissues and PDOs were fixed in 4% paraformaldehyde (PFA) for 24 h at 4 °C and embedded in paraffin according to standard protocol. Sections were subjected to H&E and immunofluorescence staining. For PBMC, cells were seeded on polylysine‐pretreated slides (Biologix, 07–2108) in the culture plates and then incubated at 37 °C to allow cells firmly adhere to the surface of the slides. After fixation and treatment with PBST containing 0.25% Triton X‐100 and 1% BSA, immunofluorescence staining was performed using the following antibodies: anti‐CD14, anti‐AFP, anti‐Vimentin, anti‐Ki67 purchased from Abcam and their corresponding secondary antibodies conjugated with fluorophores. For live cell staining, organoids were labeled with Calcein‐AM (Sigma) and then cultured on microchips. Finally, images were taken using Fluorescent Inverted Microscope (IX83, Olympus, Tokyo, Japan).

### RNA Sequencing for Transcriptome Analysis

Total RNA was extracted using Trizol reagent (Takara, Dalian, China). The mRNAs were enriched and reversely transcribed into cDNA. Then cDNA fragments were purified, end‐repaired, poly(A) added, and ligated to Illumina sequencing adapters. The ligation reaction was purified with the AMPure XP Beads(1.0X). After digestion with UNG (Uracil‐N‐Glycosylase), the products were size selected by agarose gel electrophoresis, PCR amplified, and sequenced using Illumina Novaseq6000 by Gene Denovo Biotechnology Co. (Guangzhou, China).

### Statistical Analysis

GraphPad Prism was used for figure presentation. Image J was used for organoid analysis and cell number calculation. In flow cytometry assays, the results of experimental groups were normalized to control group or presented as counts or proportion of percentage. In organoid drug tests, the results were calculated by the differences between experimental groups and control groups. All values are presented as the mean ± standard deviation (SD). The differences between two groups or among three or more groups were analyzed by unpaired two‐tailed Student's *t*‐test or one‐way analysis of variance with Bonferroni's posttest, respectively. Differences were considered statistically significant when the *p*‐value was < 0.05.

## Conflict of Interest

The authors declare no conflict of interest.

## Author Contributions

Z.Z., Z.L., and C.W. contributed equally to this work. M.W., Y.Z., and Z.Z. designed the study. Z.Z., Z.L., C.W., J.T., J.Z., J.L., K.Z., and Y.P. performed the experiments and analyzed them. M.W. and Y.Z. provided scientific expertise. Z.Z., Z.L., M.W., and Y.Z. wrote the manuscript.

## Supporting information

Supporting Information

Supplemental Movie 1

## Data Availability

The data that support the findings of this study are available from the corresponding author upon reasonable request.
