# Supplementary material for: Micro‐Engineered Organoid‐on‐a‐Chip Based on Mesenchymal Stromal Cells to Predict Immunotherapy Responses of HCC Patients
Source: Adv Sci (Weinh). 2023 Jul 23;10(27):2302640. doi: 10.1002/advs.202302640 (PMC10520686; doi:10.1002/advs.202302640)
Supplement: Supplementary file 1 — Supporting Information [file ADVS-10-2302640-s001.pdf]

ADVANCED SCIENCE
Open Access

Supporting Information

for *Adv. Sci.*, DOI 10.1002/advs.202302640

Micro-Engineered Organoid-on-a-Chip Based on Mesenchymal Stromal Cells to Predict Immunotherapy Responses of HCC Patients

Zhengyu Zou, Zhun Lin, Chenglin Wu, Jizhou Tan, Jie Zhang, Yanwen Peng, Kunsong Zhang, Jiaping Li, Minhao Wu* and Yuanqing Zhang**

Supporting Information

Micro-engineered Organoid-on-a-Chip Based on Mesenchymal Stromal Cells to Predict Immunotherapy Responses of HCC Patients

Zhengyu Zou#, Zhun Lin#, Chenglin Wu#, Jizhou Tan, Jie Zhang, Yanwen Peng, Kunsong Zhang, Jiaping Li, Minhao Wu*, Yuanqing Zhang**

# The authors contributed equally to this paper.

Supplementary Experimental section

Isolation of MSC and CAF

For MSC isolation, bone marrow aspirates were diluted in equal volumes of PBS, followed by Ficoll density gradient centrifugation as previously described[25a]. For CAF isolation, HCC tissues were digested with 0.25% Trypsin-EDTA (Biological Industries) and filtered. Then cells were seeded in culture dishes with low-glucose DMEM (GIBCO) medium containing 2 ng/ml recombinant human basic fibroblast growth factor (GIBCO). Non-adherent hematopoietic cells were removed by changing the medium every 3 days until adherent cells expanded to cover 80% of the culture dish. Then, cells were digested and reseeded at 1,000 cells/cm^2 for expanding culture. MSC/CAF at Passage 3 (P3) were assayed by flow cytometry analysis for the positive expression of CD73, CD90, CD105, CD44, CD29, and negative expression of CD45, CD31, and HLA-DR.

MSC and CAF-conditioned medium

MSC and CAF with good status were grown in a 10 cm diameter petri dish and expanded to 80%-90% full of the dish. We changed the medium to low-glucose DMEM containing no serum or cytokines and continued to culture cells for 24 to 48h. Then, the supernatant was collected, filtered with a 0.22 μm pore filter (SORFA), and stored in a -80°C refrigerator. Before use, the supernatant was thawed and mixed with RPMI 1640 complete medium or organoid culture medium at a certain proportion.

Flow cytometry

Organoids were digested with collagenase II and incubated with 0.25% Trypsin-EDTA to obtain single-cell suspensions. Staining was performed according to instruction, using the following antibodies: Brilliant Violet 421™ anti-human CD14, PerCP/Cyanine5.5 anti-human CD45, APC anti-human CD86, PE anti-human CD163, FITC anti-human CD44 and Brilliant Violet 421™ anti-human HLA-DR were purchased from Biolegend. APC anti-human CD105,

PE-Cy7 anti-human CD73, Brilliant Violet 421™ anti-human CD90, PE anti-human CD31, APC anti-human CD29, PE anti-human AFP, FITC anti-human PD-L1, and PerCP/Cyanine5.5 anti-human CD38 were purchased from BD Biosciences (San Jose, CA, USA). After fixation, cell suspensions were detected on a flow cytometer (BD FACSVerse, BD Biosciences) and analyzed with FlowJo software (Tree Star Inc., Ashland, OR, USA).

Supplementary Figure 1.

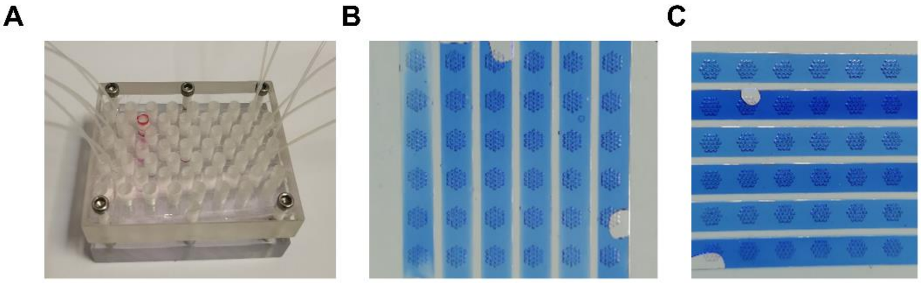

Supplementary Figure 1 Fabrication of chips using fixture. (A) Using fixture to encapsulate two-layer chip reversibly. (B) Dye filling in the chip, the bottom layer was bonded to the middle layer. (C) Dye filling in the chip, the bottom layer was bonded to the top layer.

Supplementary Figure 2.

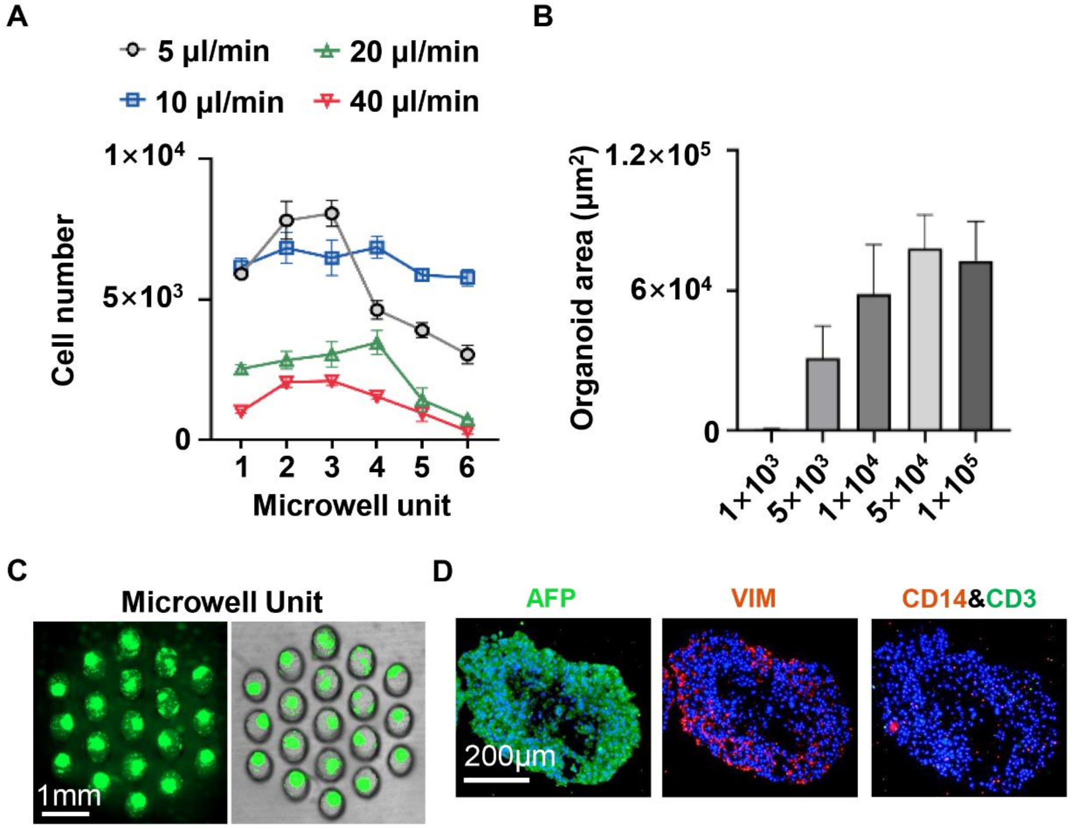

Supplementary Figure 2 Effects of flow velocities and cell density on cells distribution into microwells of microfluidic chips. (A) HepG2 cells were poured into microfluidic chips at indicated flow velocities. The total cell number in each microwell unit was analyzed by microscopy (n=3). **(B)** HepG2 cells with different cell densities were poured into the middle layer and cultured in microwells for 48h. The cell organoid area in each microwell was assessed by microscopy (n=19). **(C)** PDO was stained with Calcein-AM and poured into the middle channel at optimal velocity and cell density. Fluorescence images of the microunit were shown. **(D)** Different cell labeling in MSC-PDO-PBMC models cultured on chips. Data were shown in mean ± SD.

Supplementary Figure 3.

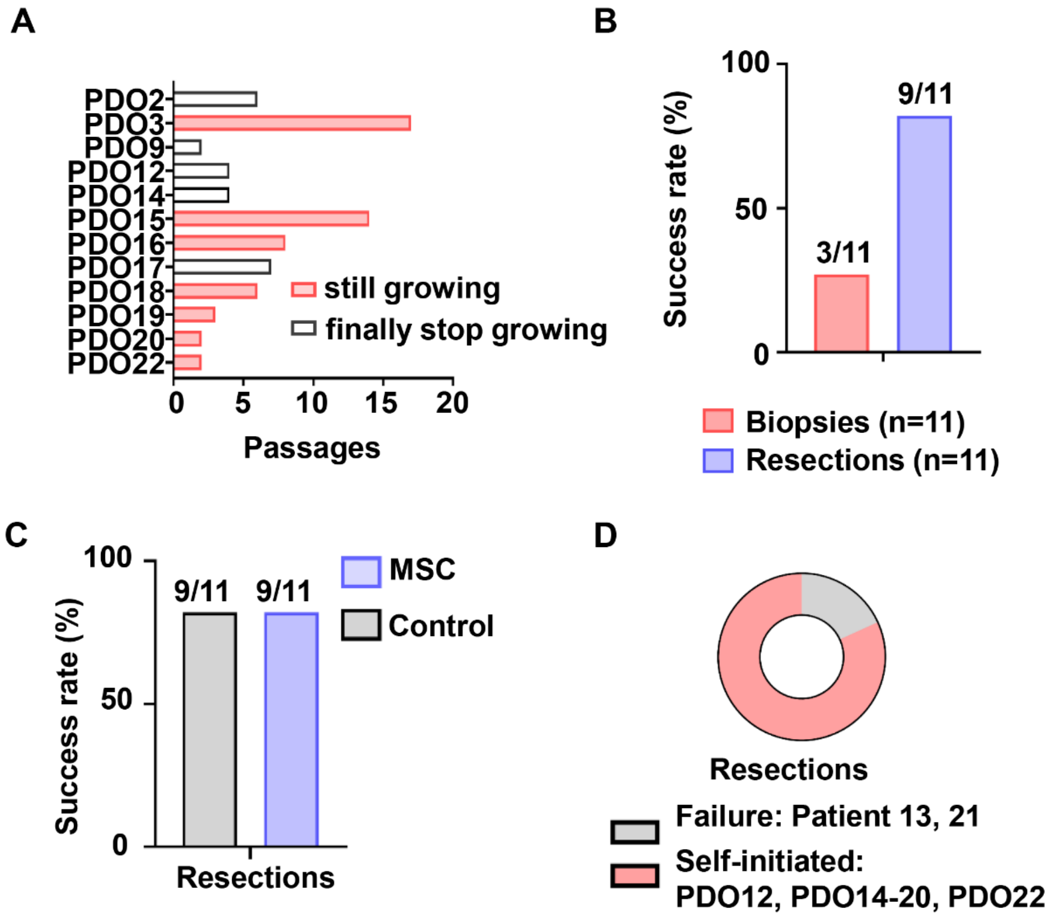

Supplementary Figure 3 HCC PDO cultured from biopsy or resection specimens. (A) PDO cultures were successfully generated from 12 out of 22 HCC patients (PDO1-11: biopsy sample, PDO12-22: resection sample) using conventional methods. Five PDOs finally stopped growing, while the other 7 PDOs kept growing, and their final or current passage numbers were shown. **(B)** Success rates of PDO culture from 11 biopsy samples or 11 resection specimens. Observation of viable tumor spheres at day 7 after culture was considered as the criteria for successfully initiated PDO cultures. **(C)** The Bar chart shows that MSC coculture did not influence the success rate of resection-derived PDO. **(D)** The pie chart shows the PDO culture results of 11 HCC resection samples (PDO12-22), in which 9 of 11 samples were self-initiated.

Supplementary Figure 4.

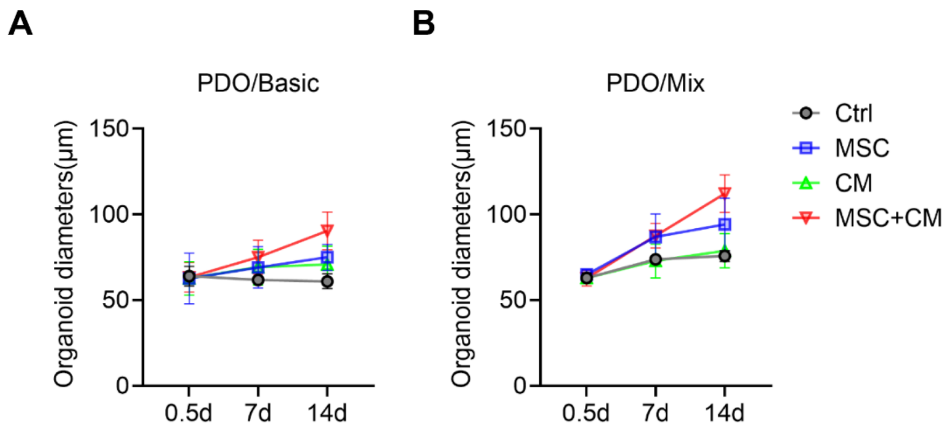

Supplementary Figure 4 MSC+CM promotes tumor growth in an organoid culture medium deficient with cytokines. (A&B) PDOs were cultured with or without MSC and/or 30%(v/v) MSC-CM, respectively, in (A) organoid culture medium without cytokines HGF, EGF, FGF, Noggin, and RSPO1 (Basic) and (B) organoid culture medium with cytokines (Mix). Their growth was recorded using microscopy. Growth curves were shown (n=5). Data were shown in mean ± SD.

Supplementary Figure 5.

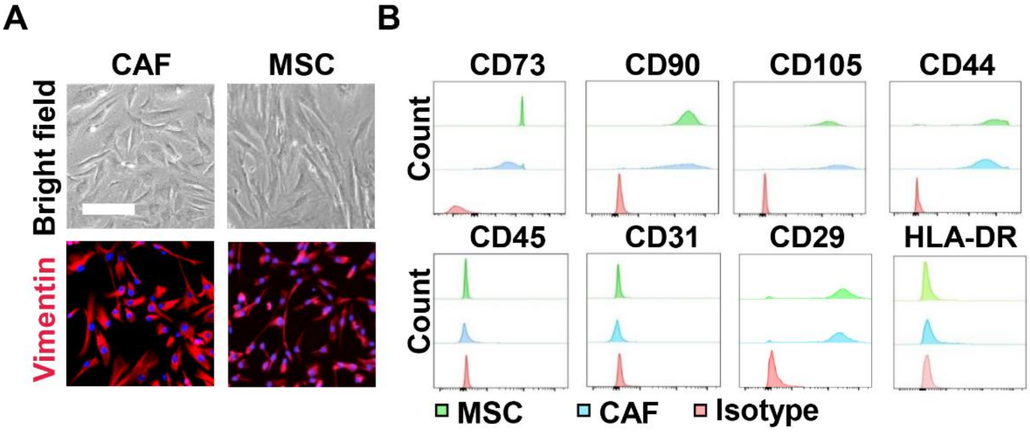

Supplementary Figure 5 MSC show the similarity of CAF. (A) Microscopy of MSC and CAF showed similar morphologies. Scale bar 100 μm. **(B)** MSC and CAF positively expressed CD73, CD90, CD105, CD29, and CD44, while CD45, CD31, and HLA-DR were negatively expressed, as assessed by flow cytometry.

Supplementary Figure 6.

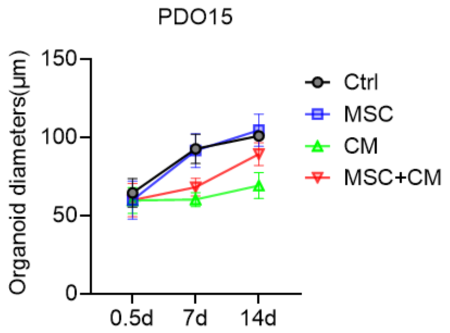

Supplementary Figure 6 MSC at Passage 10 have no tumor-promotive effects. PDOs were cultured with or without MSC after Passage 10, in the presence or absence of corresponding CM (30% v/v), and growth curves of PDOs were shown (n=5). Data were shown in mean ± SD.

Supplementary Figure 7.

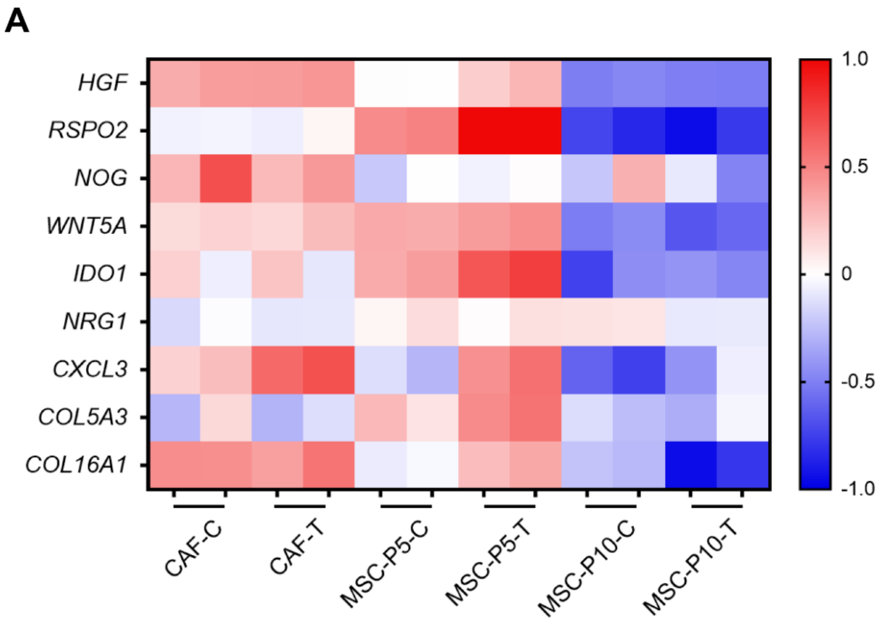

Supplementary Figure 7 Gene expression in CAF and MSC at Passage 5 and Passage 10 before (-C) or after tumor education (-T), as shown in the heatmap. Several gene expressions like *HGF*, *CXCL3*, *COL5A3*, *and COL16A1* in MSC-P5 after tumor education (MSC-P5-T) were more similar with CAF compared with control (MSC-P5-C). Tumor-educated MSC-P5 expressed higher genes of growth factors *RSPO2*, *WNT5A*, and genes of immunoregulation *IDO1*, becoming a CAF-like phenotype to promote PDO growth further and construct an immunosuppressive microenvironment.

Supplementary Figure 8.

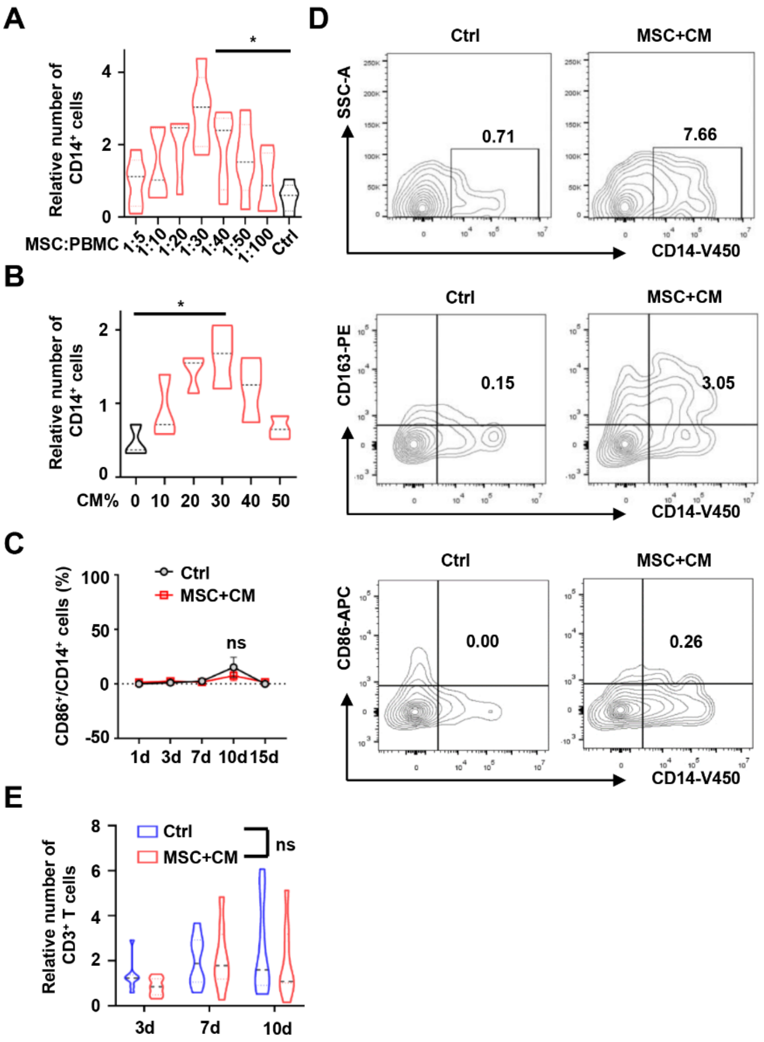

Supplementary Figure 8 MSC promotes monocyte/macrophage proliferation in ratio/dose-dependent manners. (A&B) PBMC were cocultured with MSC in different ratios of MSC: PBMC (n=5) (A) or treated with MSC-CM (n=3) (B) in different doses (%v/v). After 7 days post co-culture or treatment, the number of CD14 positive monocytes was examined by flow cytometry analysis and normalized to the original monocyte number in PBMC at day 0. **(C&D)** Flow cytometry was performed and showed that MSC promoted monocytes/macrophages and M2 differentiation and had no effect on M1 macrophage differentiation of monocytes. Data are shown as mean ± SD. ns, not significant. **(E)** PBMC were cultured in the presence of IL-2 (2000 IU/ml) and M-CSF (20ng/ml) and treated with or without MSC at a ratio (MSC: PBMC) of 1:30 plus CM (30% v/v). At 3, 7, or 10 days after treatment, the number of T cells was analyzed by flow cytometry and normalized to that on Day 0 (n=8). MSC have slightly inhibitory effects on T lymphocyte proliferation. Data are shown as mean ± SD. *p*-values were calculated with two-tailed unpaired *t*-test or one-way analysis of variance with Bonferroni's posttest. *, $p < 0.05$; ns, not significant.

Supplementary Figure 9.

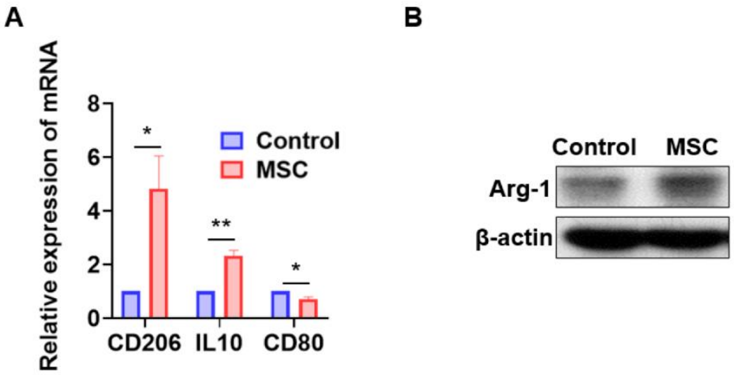

Supplementary Figure 9 MSC promote M2 differentiation of monocytes. (A) mRNA expression of indicated genes was assessed (n=3). MSC treatment for 3d promoted M2-related CD206 and IL10 expression and inhibited M1-related CD80 expression in the human monocyte cell line THP1. (B) MSC-treatment for 3d promoted M2-related Arginase-1 (Arg-1) expression in THP1 cells, as detected by Western blot. Data are shown as mean ± SD. *p*-values were calculated with two-tailed unpaired *t*-test. *, $p < 0.05$; **, $p < 0.01$.

Supplementary Figure 10.

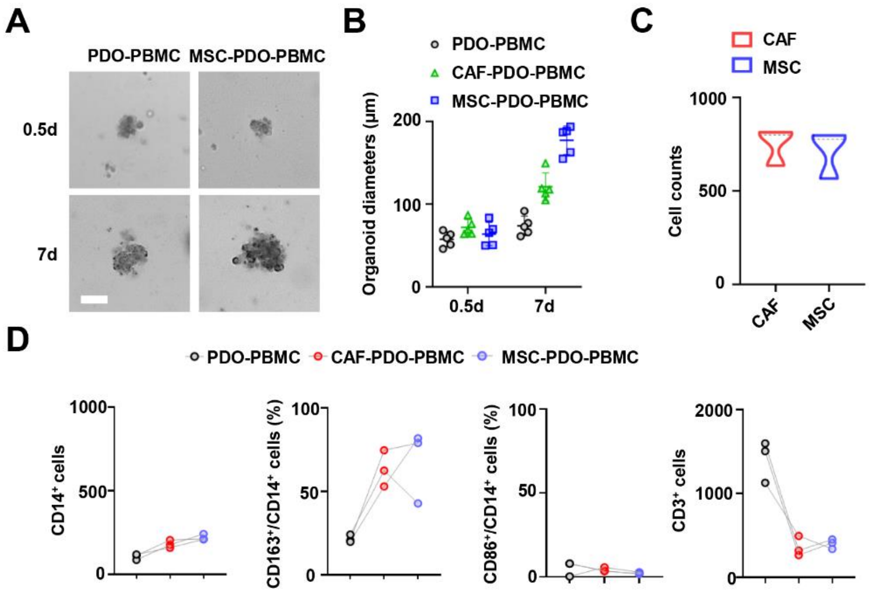

Supplementary Figure 10 Comparison of MSC and CAF in the MSC-PDO-PBMC model. (A) MSC promoted PDO growth in MSC-PDO-PBMC compared to PDO-PBMC. **(B)** PDO growth was assessed at days 0.5 and 7 when cocultured with CAF-PDO-PBMC or MSC-PDO-PBMC systems. Data were shown as mean ± SD. **(C)** MSC and CAF could be preserved and detected in MSC(CAF)-PDO-PBMC coculture systems, as assessed by flow cytometry on day 7 (n=3). **(D)** Immune cells were analyzed by flow cytometry. MSC and CAF preserved $CD14^+$ monocytes and M2 macrophages, had no effect on M1 macrophages, and inhibited $CD3^+$ lymphocyte number in MSC-PDO-PBMC coculture systems.

Supplementary Figure 11.

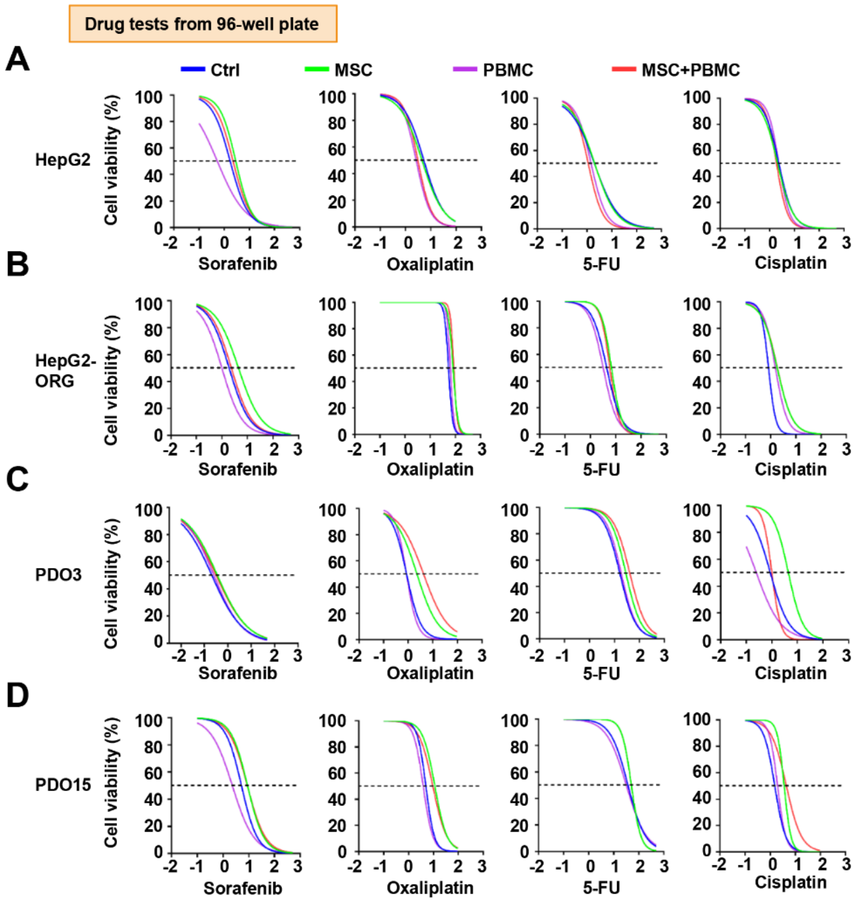

Supplementary Figure 11 Drug sensitivity tests using different PDO in well-plate. (A) HepG2 cells, **(B)** organoids, and **(C&D)** HCC PDOs were cocultured with or without MSC and/or PBMC on 96-well plates. Cell viability of these cultures in response to chemotherapeutic agents, including Sorafenib, Oxaliplatin, 5-FU, and Cisplatin, was detected by CCK8, as shown in dose-response curves. The horizontal axis was shown as log dose (μM).

Supplementary Figure 12.

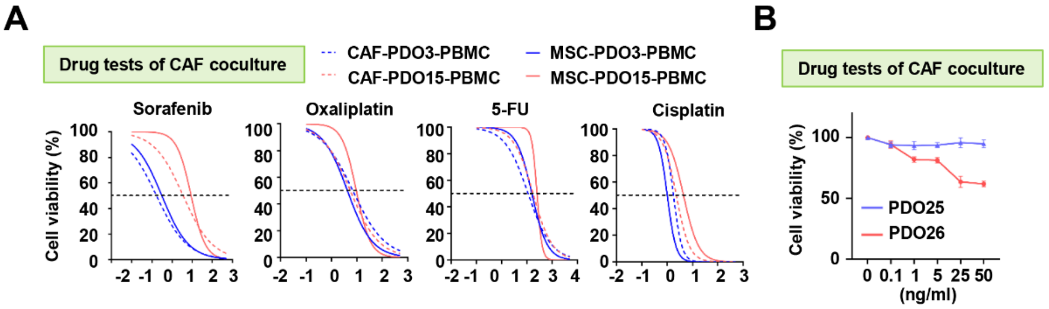

Supplementary Figure 12 Drug sensitivity tests using CAF-PDO-PBMC models. (A) Dose-response curves of PDO3 and PDO15 cocultured with CAF-PBMC to chemotherapeutic agents on 96-well plates were shown and compared with those cocultured with MSC-PDO-PBMC. The horizontal axis was shown as log dose (μM). **(B)** CAF-PDO-PBMC models of PDO25 and PDO26 were performed immunotherapy tests using Atezolizumab. PDO25 coculture models were resistant to Atezolizumab, and PDO26 coculture models responded to Atezolizumab.

Supplementary Figure 13.

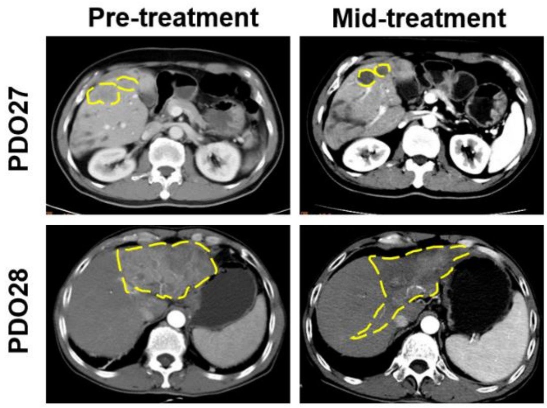

Supplementary Figure 13 CT scan images of PDO27/28 corresponding patients before and after treatment with Atezolizumab. CT scan showed that patients of PDO27 and PDO28 were respectively sensitive and resistant to Atezolizumab treatment.

Supplementary Table 1 Patient information of PDO used to establish MSC-PDO-PBMC models. PDOs from patients 23-28 were used to evaluate the drug sensitivity prediction accuracy of coculture models. Patients 23 and 24 were, respectively, Sorafenib-resistant and Sorafenib-sensitive. Patients 25/28 and 26/27 were respectively resistant and sensitive to Atezolizumab (anti-PD-L1) treatment.

ID	Age	Sex	Cirrhosis	Edmondson[a)]	BCLC[b)]	MVI[c)]
Patient1	52	Male	Yes	III	C	Yes
Patient2	43	Male	Yes	II − III	B	No
Patient3	54	Female	No	I − II	A	No
Patient4	46	Male	Yes	II − III	B	No
Patient5	65	Male	No	II	B	Yes
Patient6	37	Male	Yes	II	A	Yes
Patient7	54	Male	Yes	II	A	No
Patient8	61	Male	Yes	I − II	A	No
Patient9	53	Male	Yes	II − III	B	No
Patient10	58	Male	Yes	II − III	B	Yes
Patient11	62	Male	No	II	A	No
Patient12	67	Female	Yes	II − III	A	No
Patient13	54	Male	Yes	II	A	No
Patient14	47	Female	Yes	II	A	Yes
Patient15	59	Male	Yes	II − III	B	No
Patient16	66	Male	Yes	II − III	A	No
Patient17	60	Male	Yes	III	C	Yes
Patient18	61	Male	Yes	II-III	B	Yes
Patient19	15	Male	No	II	A	No
Patient20	48	Male	Yes	II	B	Yes
Patient21	44	Male	Yes	I-II	A	No
Patient22	52	Male	Yes	II-III	B	Yes
Patient23	63	Female	No	III	C	Yes
Patient24	42	Male	Yes	II − III	C	Yes
Patient25	45	Male	Yes	II	C	Yes
Patient26	63	Male	Yes	III	C	Yes
Patient27	60	Male	Yes	III	C	Yes
Patient28	59	Male	Yes	III	C	Yes

a) Edmondson, the Edmondson-Steiner grading; b) BCLC, the Barcelona Clinic Liver Cancer staging classification; c) MVI, microvascular invasion.

Supplementary Movie 1: Dye flowed into the top layer in the three-layer chip.
